# Comprehensive Qualitative Drug Screening in Emergency Toxicology Using an Automated LC–MS^n^ System:Simultaneous Quantification of Relevant Drugs and Metabolites in Blood Plasma

**DOI:** 10.1002/dta.3855

**Published:** 2025-01-19

**Authors:** Selina Hemmer, Maximilian Ninnig, Lea Wagmann, Sascha K. Manier, Markus R. Meyer

**Affiliations:** ^1^ Department of Experimental and Clinical Toxicology, Institute of Experimental and Clinical Pharmacology and Toxicology, Center for Molecular Signaling (PZMS) Saarland University Homburg Germany

**Keywords:** clinical toxicology, emergency toxicology, LC–MS^n^, method validation

## Abstract

Rapid and comprehensive qualitative and quantitative analytical procedures are crucial in 24/7 emergency toxicology (ET) to support diagnosis and treatment of acute intoxications and to monitor their progression and efficacy of detoxification strategies. This study aimed to develop the simultaneous qualitative and quantitative analysis of 62 drugs, as well as seven active metabolites in human blood plasma using an automated liquid chromatography (LC)‐linear ion trap mass spectrometry (MS) screening system. Sample preparation was conducted by liquid–liquid extraction, and plasma concentrations were determined using an electronically stored 5‐point calibration. Validation was performed according to international guidelines and recommendations for ET including selectivity, carry‐over, accuracy, precision, and matrix effects. Finally, applicability was evaluated using case samples and proficiency tests. The method demonstrated selectivity for all analytes, with no significant carry‐over or matrix effects. Accuracy and precision recommended for ET could be fulfilled for all tested analytes, except for 10 analytes. Patient plasma samples were analyzed and compared with results obtained by reference LC‐tandem MS or gas chromatography‐MS methods. Furthermore, the applicability of the method could be demonstrated. It provides a fast, robust, and reliable blood plasma screening for 69 analytes and an additional quantification of 59 analytes relevant in ET. The use of an electronically stored 5‐point calibration and a simplified “push and print solution” allows for straightforward assessment of blood plasma levels of the analytes.

## Graphical abstract

Simultaneous qualitative and quantitative analysis of 62 drugs and seven active metabolites in human blood plasma by automated liquid chromatography linear ion trap mass spectrometry.

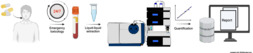

## 1 Introduction

Reliable, rapid, and cost‐effective analytical procedures are needed for 24/7 emergency toxicology (ET). The outcomes of such analyses can support clinicians in evaluating emergencies and treatment options by allowing thorough differential diagnosis and rule out acute poisoning to prevent unnecessary treatment and expenses [1, 2, 3, 4, 5]. Analyses should first include a qualitative screening procedure with confirmed identification covering a wide range of drugs [2, 3]. Second, blood levels of identified drugs should be quantified in order to assess the severity of intoxication [1, 2]. As these analyses can provide guidance for further treatment, it is essential that the duration for such analyses is kept to a minimum. Several comprehensive qualitative screening methods have been published for both blood and urine using gas chromatography (GC)‐mass spectrometry (MS) or liquid chromatography (LC) coupled to low‐ or high‐resolution MS [6, 7, 8, 9, 10, 11] and are well established in clinical and forensic toxicology. For quantification, fully validated therapeutic drug monitoring (TDM) approaches are often used, among others [12]. However, TDM approaches frequently target a specific class of drugs and are often time‐consuming, rendering them unsuitable for ET, where the analysis of a large number of drugs from different classes is required within a limited period of time. However, there are also methods available for ET that allow for screening and simultaneous or subsequent quantification. Meyer et al. developed and validated a method for blood‐level assessment of 40 relevant drugs based on an established blood‐screening procedure using 1‐point calibration and GC–MS [3]. Michely and Maurer developed a similar method using LC‐tandem MS (MS/MS) to supplement the GC–MS method [2]. Nevertheless, for both methods, the data processing was handled manually potentially entailing mistakes. Therefore, Caspar et al. developed and validated a strategy for automated quantification of several drugs and active metabolites in blood plasma based on a linear ion trap LC–MS^n^ system with standardized LC and MS settings including eluents, column, chromatographic gradient, source conditions, MS settings, and spectral database [1].

The present study aimed to extend the method by Caspar et al. via addition of further 45 analytes often requested in ET. These analytes included classes such as analgesics, anticonvulsants, antidepressants, antiemetics, antihistamines, antihypertensives, benzodiazepines, or neuroleptics. The validation should be based on international guidelines and recommendations for 24/7 emergency toxicology [13, 14]. Finally, the proof of concept and applicability should be demonstrated by analysis of proficiency tests and patient blood samples submitted to the authors' laboratory for regular toxicological analysis.

## 2 Experimental Section

### 2.1 Chemicals and Reagents

All reference standards of the drugs and metabolites were obtained from the corresponding pharmaceutical companies. The internal standards (IS) trimipramine‐d_3_ and diazepam‐d_5_ were obtained from LGC Standards (Wesel, Germany). Ammonium formate and formic acid were obtained from Merck (Darmstadt, Germany). Acetonitrile, dimethyl sulfoxide (DMSO), and methanol (all LC–MS grade) were from VWR (Darmstadt, Germany). Water was purified with a Millipore filtration unit (18.2 Ω × cm water resistance). Pooled plasma samples were obtained from a local blood bank.

### 2.2 Stock Solutions, Working Solutions, Calibrators, and Quality Controls

Stock solution at 1 or 10 mg/mL in case of carbamazepine, ketamine, oxcarbazepine, and paracetamol was freshly prepared in duplicates, once for calibrator and once for quality control solutions. All substances were solved in methanol, except for oxcarbazepine that was solved in DMSO. The analytes were split into three different mixtures (see Table S1). Their working solutions were prepared by diluting the stock solutions with methanol to a concentration 10‐fold higher than the final plasma concentration (see Table 1). All solutions were stored at −20°C. Five calibrators, 1‐point calibrator and two different quality control (QC) samples containing low and high concentrations were prepared by spiking pooled human blank plasma with the prepared working solution.

**TABLE 1:** Final plasma concentrations in ng/mL of the analytes in calibrators (Cal 1–5) and quality control samples (QC) low and high as well as the used weightings of linear calibration model and the therapeutic and toxic ranges to Schulz et al. [23].

Analyte	Weighting	Cal 1	Cal 2	Cal 3	Cal 4	Cal 5	LLOQ	QC low	QC high	Therapeutic range	Toxic above
Alprazolam	1/x^2^	50	125	250	375	500	50	60	400	5–50	100
Amisulpride	1/x^2^	100	500	1000	1500	2000	100	120	1600	100–400	640
Amitriptyline	1/x^2^	100	500	1000	1500	2000	100	120	1600	50–300	500
Aripiprazole	Equal	250	500	1000	1500	2000	250	300	1600	100–350	1000
Biperiden	1/x^2^	50	125	250	375	500	50	60	1600	1.0–6.5	13
Bisoprolol	Equal	100	500	1000	1500	2000	100	120	1600	10–100	200
Bromazepam	Equal	1000	1500	2000	2500	3000	1000	1200	2400	50–200	300
Carbamazepine	1/x^2^	2500	5000	10,000	15,000	20,000	2500	3000	16,000	2000–12,000	10,000
Chlorprothixene	Equal	250	500	1000	1500	2000	250	300	1600	20–300	400
Citalopram	1/x^2^	100	500	1000	1500	2000	100	120	1600	50–110	220
Clobazam	1/x^2^	100	500	1000	1500	2000	100	120	1600	30–300	500
Clozapine	1/x^2^	100	500	1000	1500	2000	100	120	1600	100–600	600
Codeine	1/x^2^	250	500	1000	1500	2000	250	300	1600	30–250	500
Desipramine	Equal	250	500	1000	1500	2000	250	300	1600	10–500	500
Diazepam	1/x^2^	500	1000	1500	2000	2500	500	600	2000	Sum 100–2500	Sum 5000
Diazepam‐M (nor‐)/Nordiazepam^+^	Equal	750	1000	1500	2000	2500	750	900	2000	120–800	1500
Dihydrocodeine	1/x^2^	250	500	1000	1500	2000	250	300	1600	30–250	500
Diltiazem	1/x^2^	100	500	1000	1500	2000	100	120	1600	30–250	800
Diphenhydramine	1/x^2^	500	1000	1500	2000	2500	500	600	2000	50–100	1000
Doxepin	1/x^2^	100	500	1000	1500	2000	100	120	1600	Sum 50–150	Sum 300
Doxepin‐M (nor‐)	Equal	50	125	250	375	500	50	60	400	—	—
Doxylamine	1/x^2^	200	500	1000	1500	2000	200	250	1600	50–200	1000
Flupirtine	Equal	1000	1500	2000	2500	3000	1000	1200	2400	500–1500	3000
Haloperidol	1/x^2^	50	125	250	375	500	50	60	400	1–17	50
Hydromorphone	1/x^2^	50	125	250	375	500	50	60	400	5–30	100
Imipramine	1/x^2^	250	500	1000	1500	2000	250	300	1600	50–350	500
Ketamine	1/x^2^	1000	2500	5000	7500	10,000	1000	1200	8000	100–6000	7000
Levomepromazine	1/x^2^	200	500	1000	1500	2000	200	250	1600	5–200	400
Lorazepam	1/x^2^	500	1000	1500	2000	2500	500	600	2000	20–250	300
Maprotiline	1/x^2^	100	500	1000	1500	2000	100	120	1600	75–130	500
Melperone	1/x^2^	100	500	1000	1500	2000	100	120	1600	30–100	200
Methadone	1/x^2^	100	500	1000	1500	2000	100	120	1600	50–600	600
Metoclopramide	1/x^2^	100	500	1000	1500	2000	100	120	1600	10–150	200
Metoprolol	1/x^2^	100	500	1000	1500	2000	100	120	1600	20–600	7800
Mianserin	1/x^2^	50	125	250	375	500	50	60	400	15–70	250
Midazolam	1/x^2^	100	500	1000	1500	2000	100	120	1600	40–250	1000
Mirtazapine	1/x^2^	150	500	1000	1500	2000	150	180	1600	30–300	1000
Moclobemide	Equal	500	1500	2500	4000	5000	500	600	4000	300–3000	2000
Olanzapine	1/x^2^	50	125	250	375	500	50	60	400	1–80	100
Opipramol	1/x^2^	100	500	1000	1500	2000	100	120	1600	50–500	1000
Oxazepam	Equal	1000	1500	2000	2500	3000	1000	1200	2400	200–1500	2000
Oxcarbazepine	1/x^2^	2500	10,000	20,000	40,000	80,000	2500	3000	65,000	10,000‐35,000	35,000
Oxycodone	1/x^2^	250	500	1000	1500	2000	250	300	1600	5–100	200
Paracetamol	1/x^2^	2500	30,000	60,000	90,000	120,000	2500	3000	96,000	5000‐25,000	100,000
Paroxetine	1/x^2^	50	125	250	375	500	50	60	400	2–65	400
Perazine	1/x^2^	100	500	1000	1500	2000	100	120	1600	10–230	460
Pethidine	1/x^2^	500	1000	1500	2000	2500	500	600	2000	100–800	1000
Pethidine‐M (nor‐)	Equal	50	125	250	375	500	50	60	400	—	—
Pipamperone	Equal	250	500	1000	1500	2000	250	300	1600	100–400	500
Promethazine	1/x^2^	100	500	1000	1500	2000	100	120	1600	10–200	1000
Prothipendyl	Equal	50	125	250	375	500	50	60	400	30–80	500
Quetiapine	1/x^2^	100	500	1000	1500	2000	100	120	1600	100–500	1800
Ramipril	1/x^2^	50	125	250	375	500	50	60	400	1–40	80
Risperidone	1/x^2^	50	125	250	375	500	50	60	400	Sum 20–60	Sum 120
Risperidone‐M (9‐hydroxy‐)/Paliperidone*	1/x^2^	100	500	1000	1500	2000	100	120	1600	20–60	120
Sertraline	Equal	250	500	1000	1500	2000	250	300	1600	10–500	300
Sulpiride	Equal	500	1000	1500	2000	2500	500	600	2000	50–1000	1500
Tapentadol	1/x^2^	100	500	1000	1500	2000	100	120	1600	10–300	—
Temazepam	1/x^2^	500	1000	1500	2000	2500	500	600	1600	20–900	1000
Tilidine	1/x^2^	100	500	1000	1500	2000	100	300	1600	50–300	—
Tilidine‐M (nor‐)	1/x^2^	100	500	1000	1500	2000	100	120	1600	—	—
Tramadol	1/x^2^	250	500	1000	1500	2000	250	300	1600	100–1000	1000
Tramadol‐M (*O*‐demethyl)	1/x^2^	100	500	1000	1500	2000	100	120	1600	—	—
Venlafaxine	1/x^2^	100	500	1000	1500	2000	100	120	1600	Sum 100–400	Sum 1000
Venlafaxine‐M (*O*‐demethyl)	1/x^2^	100	500	1000	1500	2000	100	120	1600		
Verapamil	Equal	100	500	1000	1500	2000	100	120	1600	10–400	1000
Zolpidem	1/x^2^	100	500	1000	1500	2000	100	120	1600	80–200	500
Zopiclone	Equal	100	500	1000	1500	2000	100	120	1600	10–120	150
Zuclopenthixol	1/x^2^	50	125	250	375	500	50	60	400	4–100	100

*Note:* Sum = concentration of drug plus active metabolite; + = Nordiazepam possible parent drug or metabolite of diazepam; * = paliperidone (9‐hydroxyrisperidone) possible parent drug or metabolite of risperidone.

### 2.3 Blood Plasma Preparation

In accordance with minor deviations from previous published procedures [1, 2, 3, 15, 16], a liquid–liquid extraction (LLE) for sample preparation was used. One milliliter of blood plasma was mixed with 100 μL of trimipramine‐d_3_ (0.1 mg/mL) as IS, 2 mL saturated aqueous sodium sulfate solution and 5 mL diethyl ether‐ethyl acetate mixture (1:1, *v/v*). The mixture was shaken manually for 20 s and centrifuged for 2 min at 3000× *g* at 24°C. The upper solvent phase was transferred into a pointed flask. Second, a volume of 500 μL sodium hydroxide (1 mol/L, pH 8–9) and 5 mL diethyl ether‐ethyl acetate mixture (1:1, *v/v*) was added to the remaining liquid and again mixed manually for 20 s, followed by 2 min of centrifugation at 3000× *g* at 24°C. Upper solvent phase was again transferred into the same pointed flask and evaporated to dryness at 70°C. The combined residues were reconstituted in 100 μL methanol. A 20 μL aliquot was then diluted with 80 μL of a mixture (9:1, *v/v*) eluent A (2 mM aqueous ammonium formate containing 0.1% formic acid and 1% acetonitrile) and eluent B (acetonitrile containing 0.1% formic acid with 2 mM ammonium formate and 1% water), followed by 2 min of centrifugation at 21,130× *g* at −10°C. Five microliters of the supernatant were injected onto the LC–MS^n^ system under the conditions described below.

### 2.4 LC‐MS^n^ Settings

According to a published procedure [1], the LC‐MS^n^ system consisted of a Dionex UltiMate 3000 LC‐system (Thermo Fisher Scientific, TF, Dreieich, Germany) and an amaZon speed ion trap mass spectrometer (Bruker Daltonik) coupled to an electrospray ionization (ESI) source. Gradient elution was performed on a TF Acclaim 120 C_18_ column (100 mm × 2.1 mm, 2.2 μm) using 2 mM aqueous ammonium formate containing 0.1% formic acid and 1% acetonitrile (eluent A) and acetonitrile containing 0.1% formic acid with 2 mM ammonium formate and 1% water (eluent B). Gradient and flow rate were set as follows: 0–1 min hold 1% B; 1–8 min linear increase to 95% B; 8–9 min hold at 95% B; 9–11 min hold at 1% B at constant flow rate of 0.5 mL/min. The injection volume was set to 5 μL.

The MS was used in AutoMS^n^ mode using UltraScan in positive and negative ionization switching mode with mass range from *m/z* 70 to 800 at 32,500 *m/z*/s; ICC target, 70,000; max. Accu time, 50.00 ms. AutoMS^n^ spectra were generated up to *n* = 3, if possible. Data‐dependent acquisition was performed to record MS^2^ and MS^3^ spectra according to a scheduled precursor list (SPL) containing the retention time of each analyte, and the nominal mass of the respective precursor ions for all MS stages and active exclusion was set to exclude after 1 spectra and release after 0.50 min. The following ESI parameters were used: capillary voltage, 4500 V; end plate offset, 500 V; nebulizer gas, 29.0 psi; dry gas, 10 L/min; dry temp., 320°C. The data were automatically processed, and compounds were identified via the unmodified TT library (version Toxtyper 2_0 Library, Bruker Daltonik). A result report as pdf was automatically generated by the DataAnalysis software (Version 4.4, Bruker Daltonik).

### 2.5 Ionization Effects of Co‐Eluting Analytes

Ion suppression and enhancement of co‐eluting analytes was tested according to published procedures [1, 2, 17]. One set of all analytes and IS in one mixture at a concentration of 1 mg/L and one set of single analyte solutions including trimipramine‐d_3_ were analyzed in triplicates and the IS‐normalized peak areas using diazepam‐d_5_ were compared. The acceptance criteria (AC) for ion suppression and enhancement were set to ± 25% [17].

### 2.6 Method Validation

Method validation was done according to the “Guideline on bioanalytical method validation” of the European Medicines Agency (EMA) [13] and the “Recommendations of criteria for development and validation of analytical methods for estimating concentrations of drugs in blood to be used in 24/7 clinical toxicology” of the Society of Toxicological and Forensic Chemistry (GTFCh) [14]. Statistical evaluation was performed using Microsoft Excel (version 16.84, Redmond, WA, USA), GraphPad Prism 9.00 (GraphPad Software, La Jolla, CA, USA), and R (version 2022.12.0+353) [18].

#### 2.6.1 Selectivity and Carry‐Over

Selectivity was tested by analyzing drug‐free human blood plasma samples from six donors. Carry‐over was tested after the injection of the highest calibrator (calibrator 5) followed by blank blood plasma sample. According to the EMA guideline, absence of interfering compounds at the corresponding retention times of the analytes and carry‐over in a blank blood plasma sample are accepted where the response is less than 20% of the lower limit of quantification (LLOQ) for analytes and 5% for IS [13].

#### 2.6.2 Linearity of the Calibration Curves and Limits of Quantification

For calibration curve, five calibrators were prepared by spiking the working solutions in pooled blank plasma at a ratio of 1:10 followed by an LLE as described above. Calibrator 1 and 5 were defined as concentrations for LLOQ and upper limit of quantification (ULOQ) for all analytes, respectively.

The determination of linearity was done using a manually programmed R script (https://github.com/sehem/Calibration_Weighting.git). The linearity was tested according to Mandel. Therefore, the respective squares of the residual standard deviations from linear and quadratic regression were used and *p*‐value was set to 0.05. Linear regression with several weightings (equal, 1/x, 1/x^2^, 1/y, 1/y^2^) were tested by fitting three individual curves of each analyte. Suitability of the models was evaluated using the sum of square residuals (SSR) by choosing the model with the lowest SSR and calculation of the residual sum of squares. The lowest sum of squares indicated the best weighting model (*F*‐test heteroscedasticity). According to the EMA guideline, the acceptance criteria (AC) for back calculation of each calibrator should be within ± 15% and ± 20% for LLOQ, respectively. At least 75% of the calibration standards must fulfill these criteria [13], which means four out of five in case of the current method.

#### 2.6.3 Matrix Effects

Matrix effects were done according to the GTFCh recommendations [14, 19]. Therefore, two different sets of samples were prepared at QC low and QC high in six replicates. Sample set 1 contained neat analyte solution in methanol. Sample set 2 represented blank plasma from six individual donors spiked with analyte solution after extraction. For validation, the matrix factor was calculated by comparing sample set 2 to sample set 1. According to the GTFCh, the AC were set to matrix effects within ± 30% [14].

#### 2.6.4 Accuracy and Precision

Within‐run accuracy and precision were performed by analyzing five samples at three different concentration levels (LLOQ, QC low, and QC high). Between‐run accuracy and precision were performed by analyzing five samples from three different concentration levels mentioned before on at least three different days. The AC for the validation in emergency toxicology according to the GTFCh guideline were set to ± 30% of the nominal value of the QCs, which correspond to the mean concentration of the defined concentration level for both within‐ and between‐run accuracy. For within‐ and between‐run precision, the CV had to be ≤ 30% [14].

### 2.7 Stability of Electronically Stored Calibration

To assess the stability of an electronically stored calibration, freshly prepared QC high extracts were analyzed over a 2‐year period. The electronically stored calibration was based on calibrators 1–5 analyzed in triplicates on the first day. The mean slopes and intercepts of each analyte were calculated using GraphPad Prism 9.00 (GraphPad Software, La Jolla, CA, USA) and transferred to a CSV file (see Table S2). The processing method is conducted using the aforementioned CSV file in order to quantify the analytes included in this study. Subsequently, the stability of the electronically stored calibration was monitored by analyzing one freshly prepared QC high sample every second week. The calculated concentrations via the stored calibration were compared with the nominal concentration levels of the QC high. The AC were set to ± 30% in accordance with the recommendations for emergency toxicology [14]. Additionally, 1‐point calibrators were used, with concentrations aligned with those of calibrator 3 (see Table 1). In the event of a decrease in instrument performance, 1‐point calibrators can be used for time saving reasons to adjust the slope of the stored calibration and thus ensure continuous reliability. For this purpose, freshly prepared 1‐point calibrators were analyzed in triplicates and evaluated data were automatically transferred to calibration information CSV file (see Table S2).

### 2.8 Proof of Concept and Applicability Studies

A total of 29 blood plasma samples submitted to the authors' laboratory for regular toxicological analysis and several interlaboratory tests were analyzed for proof of concept studies. Results were compared with the acceptance range of the interlaboratory tests or in case of patient samples with results obtained by reference methods using LC‐tandem MS or GC–MS [2, 3].

## 3 Results and Discussion

The objective of this study was to simultaneously identify and semiquantify selected analytes relevant in a 24/7 ET setting. The selection of analytes within this study was based on their relevance in ET and the frequency of request in the authors' laboratory over the past 10 years and aimed to complement those included in the paper by Caspar et al. [1]. This expansion by adding 45 analytes, deemed the need for a (re‐)validation of the procedure including proof of concept studies as, for example, the EMA mandates re‐validation when substantial alterations are made to a method, including the expansion of analyte panels [13].

The method was thus validated based on recommendations of the GTFCh [14] and EMA [13]. Some parameters were not investigated as they are not of relevance in ET such as stability of processed samples. Samples are analyzed immediately after preparation and are not stored in the autosampler. Furthermore, recovery was not evaluated, as the employed extraction method has been comprehensively validated in prior studies [1, 2, 3]. Additionally, the method focuses on the reliable determination of toxic concentrations, and minor losses during extraction have no significant impact on the method's applicability or the clinical interpretation of the results.

### 3.1 Method Development and Validation

#### 3.1.1 Ionization Effects of Co‐Eluting Analytes

**FIGURE 1:**
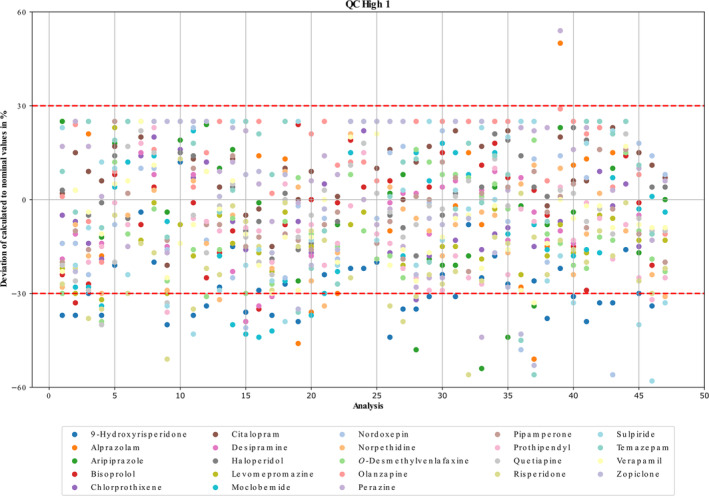
Reconstructed ion chromatograms of all analytes generated by DataAnalysis after analysis of the high‐concentrated quality control sample (QC High).

As the TT system consists of a fixed combination of LC and MS components and settings, eluents, column, chromatographic gradient, MS conditions, and SPL have not been modified. The chromatographic separation within 11 min allows sufficient fast analysis in an ET setting but does in turn not allow baseline separation of all 69 analytes (Figure 1). Therefore, ionization suppression or enhancement effects of co‐eluting analytes were tested by comparing the IS‐normalized peak areas from a set containing all analytes in one mixture to a set of single analyte solution. The results revealed that distributing the analytes into three working solutions was necessary to minimize ionization effects due to co‐elution (Table S1).

#### 3.1.2 Selectivity and Carry‐Over

Analytes and internal standards need to be distinguished from endogenous substances and other drugs. Thus, identification of the analytes was based on their retention times (range ± 0.4 min), their MS^2^ and/or MS^3^ spectrum using the TT library and published standard setting [1, 20, 21, 22]. Possible interferences were evaluated using blood plasma samples from six different donators known to be free of the analytes being tested. For all six samples, no interfering signals or false positive results were observed.

In addition, possible analyte carry‐over was investigated by injection of pooled blank plasma samples after the highest calibrator concentration (calibrator 5) to exclude any transfer from one sample to another. No analyte carry‐over was observed, but it should be recommended to analyze blank samples after any intoxication to prevent possible carry‐over from sample concentrations above the tested concentrations.

#### 3.1.3 Linearity of Calibration and Limits of Quantification

The concentration ranges of the drug levels were selected in the method to cover the upper therapeutic and toxic range according to the values reported by Baselt and Schulz et al. [23, 24].

The range of calibration for each analyte was set to be applicable for use in ET, meaning that concentrations below the therapeutic range or within were not of importance, as the purpose of the method should be to identify possible intoxications. Thus, the upper therapeutic range served as reference point for the lower limit of quantification (LLOQ) in most cases, with minor adjustments for low‐dosed analytes, such as biperiden, haloperidol, hydromorphone, ramipril, and risperidone, or such with low MS response, such as bromazepam and oxycodone. For those analytes, quantification was only possible for higher concentration. Regarding the upper limit of quantification (ULOQ), the concentration was set to 2000 ng/mL for most analytes, except for the high‐dosed compounds carbamazepine, ketamine, oxcarbazepine, and paracetamol.

All tested analytes could be fitted into a linear calibration model using different weighting factors (equal, 1/x, 1/x^2^, see Table 1). The back calculation of the calibrators for all analytes was within the ranges defined by the EMA guideline [13].

#### 3.1.4 Matrix Effects

The presence of co‐eluting matrix components can reduce or enhance the ion intensity of analytes, affecting the reproducibility and accuracy of the method. Therefore, the presence or absence of matrix effects in biological matrix was investigated. Matrix effects and corresponding CVs for each analyte are shown in Table S3. All analytes were within the acceptable range of ± 30% matrix effect.

#### 3.1.5 Accuracy and Precision

Quantification was automatically done by integrating the peak area in full scan of the protonated precursor mass of the analytes and corresponding IS. The peak area ratio of each analyte to the IS was compared with an electronically stored 5‐point calibration to calculated plasma concentrations. Analytes and calibration information were stored in a CSV file (Table S2). The file contains the analytes names, the used IS (trimipramine‐d3), the slope and intercept of the calibrations curve, the limits of quantification (LLOQ and ULOQ), the concentration unit (ng/mL), the calibration concentration (1‐point calibration), and in case the *m/z* value of the quantifier ion. Results are shared via an automatically generated PDF report.

Results for within‐run and between‐run accuracy and precision are shown in Tables S4 and S5, respectively. According to the recommendations of the GTFCh, blood level determinations in the context of 24/7 emergency toxicology allow a deviation of ± 30%. Regarding within‐run accuracy and precision, most analytes were within the GTFCh criteria of mean concentration within ± 30% of nominal concentration and precision did not exceed a CV of ± 30% except aripiprazole, diphenhydramine, ketamine, melperone, pethidine, sertraline, tilidine, and tilidine‐M (nor‐). In addition to the previously mentioned analytes, olanzapine and pethidine‐M (nor‐) also exceeded prescribed limits for between‐run accuracy and precision. Moreover, a reliable signal could not be detected for doxylamine at the LLOQ in every run. The method implemented in this study did not consider the integrity of dilution, as surpassing the ULOQ implies severe intoxications in all instances and precise measurements exceeding the ULOQ are neglectable in clinical toxicology.

#### 3.1.6 Stability of the Electronically Stored Calibration Over a 2‐Year Period

**FIGURE 2:**
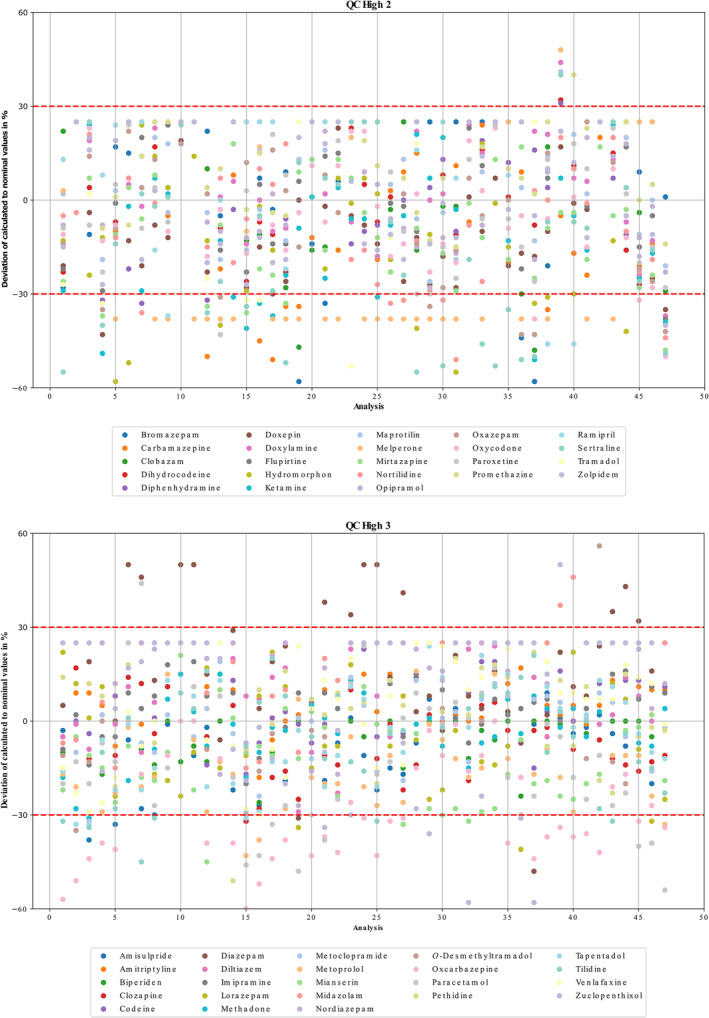
Deviations of the calculated from the nominal values of quality control samples (QC) high 1, high 2, and high 3, divided into three working solutions (1, 2, and 3) calculated based on stored 5‐point calibration over a two‐year period (in total 48 analyses). Acceptance criteria were set to ± 30%.

As the present method was developed for 24/7 toxicology applications, an electronically stored calibration was used as already described by Caspar et al. [1]. To monitor the stability of the electronically stored calibration and to extend data on accuracy and precision, freshly extracted QC high samples were measured over a two‐year period. Figure 2 shows the deviations of the calculated concentrations from the nominal values.

Among the 10 analytes that initially failed to meet the criteria mentioned above, only melperone still did not meet the criteria. The remaining nine analytes were detected and quantified with varying reliability over the period mentioned. Even among analytes that passed validation, some outliers were observed, highlighting that method validation reflects only a snapshot in time. These findings underscore the importance of regularly verifying calibration using freshly prepared or certified QC samples to ensure method reliability over extended periods.

If an analyte failed the acceptable limits of ± 30% of nominal concentration in two continuous measurements, a freshly prepared 1‐point calibration was performed to adjust the slope. The use of a fast 1‐point calibration reduces the workload and turn‐around time without loss of quality [3]. If the analyte still remains outside the acceptable limits after the slope adjustment, a new 5‐point calibration is prepared and applied to the respective analyte. Therefore, for the electronically sorted calibration, it was shown that a 5‐point calibration remains stable for an average of 6 months if supplemented by a freshly extracted 1‐point calibration every 4–8 weeks. This process, managed via separate processing method, automatically transfer 1‐point evaluation data to a CSV file (Table S2) and averages the slopes with existing entries. When a new 5‐point calibration is created, the older CSV file is archived, and a new one is initiated.

### 3.2 Proof of Concept and Applicability Studies

Proof of concept and applicability studies were done by analyzing 29 plasma samples and comparing the calculated plasma levels to those obtained by the reference GC–MS method described by Meyer et al. or the reference LC–MS/MS method by Michely and Maurer depending on the analyte [2, 3]. The results are given in Table S6. For most analytes, the quantitative results by the presented method were in accordance with those obtained by the reference methods. Although tilidine‐M (nor‐) did not fulfill all validation criteria, the calculated value in the patient plasma sample matched the reference value.

Applicability was also tested by analyzing several interlaboratory tests. The results in Table 2 were compared with the specified acceptance ranges given by Arvecon (https://www.arvecon.de/en/) and the Referenzinstitut für Bioanalytik (RfB, https://www.rfb.bio/cgi/switchLang?lang=en). Most of the interlaboratory tests refer to quantification in the field of therapeutic drug monitoring during daily routine analysis. However, the present method was primarily conceived to cover the upper therapeutic to toxic range. Thus, although most of the target values were below the LLOQ of the present method, the qualitative detection of the analytes in the therapeutic range could be demonstrated. Nevertheless, for olanzapine, which did not fulfill all validation criteria method, the values obtained were within the acceptance range. This is sufficient for the fast and reliable processing of samples during the 24/7 ET service, as it is often necessary to assess whether the drugs are in the therapeutic, overdose, or toxic range.

**TABLE 2:** Results of analyses of proficiency tests (PT) with comments if the calculated concentration passed or failed the acceptance range for PT.

Case type	Analyte	LLOQ	Calculated conc.	Target value	Accepted range	Comment
PT for vitamins and analgesic	Paracetamol	2500	21,531	18,400	12,800–24,000	Passed
			66,247	82,100	57,400–107,000	Passed
			52,800	44,200	30,900–57,500	Passed
PT for benzodiazepines	Alprazolam	50	67	53	26.6–79.4	Passed
			136	96.8	52.6–141	Passed
			79	62.9	32.3–93.5	Passed
	Bromazepam	1000	< 1000	174	100–248	Passed (< LLOQ)
			< 1000	168	96–240	Passed (<LLOQ)
	Clobazam	100	134	209	123–295	Passed
			275	298	182–414	Passed
			287	265	161–369	Passed
	Diazepam	500	752	430	272–588	Failed
			< 500	309	191–427	Passed (< LLOQ)
	Diazepam‐M (nor‐)	750	< 750	223	133–313	Passed (< LLOQ)
			< 750	337	209–465	Passed (< LLOQ)
			< 750	518	334–702	Passed (< LLOQ)
	Lorazepam	500	< 500	172	100–244	Passed (< LLOQ)
			< 500	150	86–214	Passed (< LLOQ)
			< 500	147	83–211	Passed (< LLOQ)
	Midazolam	100	164	162	92–232	Passed
			182	175	101–249	Passed
			259	204	120–288	Passed
	Oxazepam	1000	< 1000	360	224–496	Passed (< LLOQ)
			1271	345	215–475	Failed
			< 1000	336	208–464	Passed (< LLOQ)
	Temazepam	500	< 500	108	58–158	Passed (< LLOQ)
			< 500	59.5	30.3–88.7	Passed (< LLOQ)
			< 500	50.1	24.9–75.3	Passed (< LLOQ)
	Zolpidem	100	228	209	123–295	Passed
			154	128	72–184	Passed
			212	168	96–240	Passed
	Zopiclone	100	nd	37.9	17.9–57.9	Failed (< LLOQ)
			nd	58.9	29.9–87.9	Failed
PT for neuroleptics 1	Amisulpride	100	103	138	78–198	Passed
			241	295	181–409	Passed
			135	127	71–183	Passed
			274	238	142–334	Passed
	Chlorprothixene	250	< 250	93.9	50.9–136.9	Passed (< LLOQ)
			< 250	257	155–359	Passed (< LLOQ)
			< 250	90.9	49.1–132.7	Passed (< LLOQ)
			270	246	148–344	Passed
	Clozapine	100	359	445	283–607	Passed
			154	188	110–266	Passed
			430	435	277–593	Passed
			152	215	127–303	Passed
	Levomepromazine	200	nd	67.4	35.0–99.8	Failed (< LLOQ)
			< 200	177	103–251	Passed (< LLOQ)
			< 200	58.7	29.9–87.5	Passed (< LLOQ)
			< 200	159	91–227	Passed (< LLOQ)
	Olanzapine	50	< 50	39.4	18.8–60	Passed (< LLOQ)
			89	82.6	44–121.2	Passed
			< 50	49.4	24.4–74.4	Passed (< LLOQ)
			87	77.9	41.1–114.7	Passed
	Perzine	100	117	85.8	46–125.6	Passed
			346	391	245–537	Passed
			173	85.4	45.8–125	Failed
			575	410	258–562	Failed
	Promethazine	100	< 100	70.6	36.8–104.4	Passed (< LLOQ)
			150	156	88–224	Passed
			135	126	70–182	Passed
			229	254	154–354	Passed
	Quetiapine	100	< 100	44.2	21.4–67	Passed (< LLOQ)
			210	210	124–296	Passed
			< 100	59	30–88	Passed (< LLOQ)
			221	190	110–270	Passed
	Risperidone	50	< 50	7.92	3.16–12.68	Passed (< LLOQ)
			< 50	10.3	4.1–16.5	Passed (< LLOQ)
			< 50	9.13	3.65–14.61	Passed (< LLOQ)
			< 50	14.3	5.7–22.9	Passed (< LLOQ)
	Risperidone‐M (9‐hydroxy‐) /Paliperidone	100	< 100	31	14.2–47.8	Passed (< LLOQ)
			144	97.6	53.2–142	Failed
			< 100	36.8	17.4–56.2	Passed (< LLOQ)
			110	106	58–154	Passed
PT for neuroleptics 2	Aripiprazole	< 250	364	174	100–248	Failed
			311	124	68–180	Failed
			< 250	214	126–302	Passed (< LLOQ)
			nd	341	211–471	Failed
	Haloperidol	50	< 50	4.74	1.88–7.6	Passed (< LLOQ)
			< 50	13.4	5.2–21.6	Passed (< LLOQ)
			< 50	4.14	1.64–6.64	Passed (< LLOQ)
			< 50	12.8	5.0–20.6	Passed (< LLOQ)
	Melperone	1000	< 1000	61.3	31.3–91.3	Passed (< LLOQ)
			< 1000	283	173–393	Passed (< LLOQ)
			< 1000	116	64.0–168	Passed (< LLOQ)
			< 1000	242	146–338	Passed (< LLOQ)
	Pipamperone	250	< 250	134	74–194	Passed (< LLOQ)
			< 250	321	199–443	Passed (< LLOQ)
			< 250	171	99.0–243	Passed (< LLOQ)
			432	331	205–457	Passed
	Prothipendyl	50	nd	2.54	1.00–4.08	Failed (< LLOQ)
			< 50	36.8	17.4–56.2	Passed (< LLOQ)
			71	48.1	23.7–72.5	Passed
			179	119	65.0–173	Failed
	Sulpiride	500	< 500	279	111–447	Passed (< LLOQ)
			1326	537	347–727	Failed
			674	248	150–346	Failed
			1513	502	322–682	Failed
	Zuclopenthixol	50	< 50	17.3	7.1–27.5	Passed (< LLOQ)
			52	38.3	18.1–58.5	Passed
			< 50	8.49	3.39–13.59	Failed
			nd	30.3	12.1–48.5	Passed (< LLOQ)
PT for antidepressants 1	Citalopram	100	< 100	62.3	31.9–92.7	Passed (< LLOQ)
			135	115	63–167	Passed
	Mianserin	50	< 50	43.2	21–65.4	Passed (< LLOQ)
			113	91.3	49.3–133.3	Passed
	Mirtazapine	150	< 150	35	16.4–53.6	Passed (< LLOQ)
			< 150	73.9	38.7–109.1	Passed (< LLOQ)
	Paroxetine	50	126	76.7	40.5–112.9	Failed
			91	100	54–146	Passed
	Sertraline	250	< 250	60.3	30.7–89.9	Passed (< LLOQ)
			< 250	121	67–175	Passed (< LLOQ)
	Venlafaxine	100	< 100	64.7	33.3–96.1	Passed (< LLOQ)
			142	169	97–241	Passed
	Venlafaxine‐M (*O*‐demethyl‐)	100	< 100	61.8	31.6–92	Passed (< LLOQ)
			< 100	108	58–158	Passed (< LLOQ)

*Note:* Concentrations are given in ng/mL.

Abbreviation: nd, not detected.

### 3.3 Advantages and Limitations of the Method

The presented LC–MS^n^ method offers advantages, particularly in the context of emergency toxicology. The method allows for the rapid assessment of potentially toxic substances by combining qualitative identification and quantitative determination within a single run. This is crucial to support rapid clinical decisions in acute poisoning cases [1]. Furthermore, the automated data processing and reporting system optimizes the workflow by minimizing the potential for human error and providing reliable results in a short time frame, even in high‐throughput laboratory settings. Another benefit of the method is its extensive range of analytes that are covered. The method was successfully validated for 59 commonly requested drugs and metabolites, representing a wide range of toxicologically relevant substances frequently encountered in emergency toxicology. This comprehensive scope ensures that laboratories can reliably detect and quantify substances associated with acute intoxications, thus meeting the practical demands of clinical toxicology. Of particular importance is the optimization of the method for toxic concentration ranges, which are of primary concern in emergency situations where identifying overdoses or toxic exposures is more relevant than measuring therapeutic levels [1, 2, 3].

Nevertheless, in addition to these advantages, the method also presents certain limitations that should be discussed. One limitation is its sensitivity, which is inferior to that of high‐sensitivity methods based on, for example, LC–MS/MS. This diminished sensitivity results in elevated lower limits of quantification, which, in certain instances, exceed the therapeutic range of specific analytes. For example, the therapeutic range for biperiden is reported to be 1.0–6.5 ng/mL, with toxic concentrations beginning at 13 ng/mL. However, the lowest calibration point (Cal 1) was set at 50 ng/mL, which exceeds the upper limit of the therapeutic range. Consequently, the method is unable to accurately quantify concentrations within the therapeutic range for such compounds. This limitation restricts the suitability of the LC–MS^n^ method for TDM or applications requiring precise quantification at low concentration levels. Nevertheless, despite these limitations, the method remains suitable for its intended purpose, namely, the rapid and reliable detection of toxic concentrations in acute poisoning cases with some known exceptions. It fulfills the critical requirements of emergency toxicology, where timely identification of overdoses can directly impact patient outcomes and clinical decision‐making.

## 4 Conclusions

The present method offers a reliable and rapid screening of 69 analytes and additional quantification in blood plasma applicable in 24/7 emergency toxicology. It has been successfully integrated into the TT screening solution and has been validated in accordance with international guidelines and recommendations. A total of 59 out of the 69 analytes fulfilled all validation criteria and the remaining analytes can still be estimated in their quantity. The simple and fast sample preparation, short gradient, and automatic and fast quantification within 30 min based on an electronically stored 5‐point calibration contribute to the efficiency and sustainability of the method. However, it should be noted that it is only suitable for the identification of an acute overdosing.

## Author Contributions

**Selina Hemmer:** writing–original draft, formal analysis, data curation, conceptualization. **Maximilian Ninnig:** writing–review and editing, formal analysis, data curation. **Lea Wagmann:** writing–review and editing, conceptualization. **Sascha Manier:** formal analysis, writing–review and editing. **Markus R. Meyer:** writing–review and editing, resources, project administration, conceptualization.

## Conflicts of Interest

The authors declare no conflicts of interest.

## Supporting information


**Table S1.** Ionization effects using 1 mg/L solutions and internal standard diazepam‐d5. The table summarized the retention time (RT) of each analyte in minutes (min), the relative deviations of ionization in pooled solutions to single analyte solution, and the distribution of the analytes into three working solutions (1, 2, or 3). Acceptance criteria (AC) for ionization effects are ±25%. Values out of AC are marked in red.
**Table S2.** Example table for the electronically stored five‐point calibration. Analytes are listed in alphabetical order. The table contains the name of the analytes according to the Toxtyper library, the internal standard (ISTD), the slope and intercept of the five‐point calibration curves, the limits of quantification (LLOQ and ULOQ), the concentration unit in ng/mL, the calibration concentration of the one‐point calibrator, the quantifier’s *m/z* value (Quan *m/z*), and the determined slope from an one‐point run (Cal_Slope_1).
**Table S3.** Matrix effects (ME) with corresponding coefficient of variation (CV) for quality control sample (QC) Low and High. Acceptance criteria for ME were within ±30%.
**Table S4.** Within‐run accuracy and precision data. Acceptance criteria (AC) for accuracy, ±30% of nominal value and AC precision, coefficient of variation (CV) < 30%. Values out of AC are marked in red.
**Table S5.** Between‐run accuracy and precision data. Acceptance criteria (AC) for accuracy, ±30% of nominal value and AC precision, coefficient of variation (CV) < 30%. Values out of AC are marked in red.
**Table S6.** Results of analyses of plasma samples analyzed by reference LC–MS/MS [1] or GC–MS [2] methods with interpretation of the values according to Schulz et al. [3]. Concentrations are given in ng/mL; not available, −.

## Data Availability

The data that support the findings of this study are available from the corresponding author upon reasonable request.
