# Supplementary material for: Comprehensive Qualitative Drug Screening in Emergency Toxicology Using an Automated LC–MSn System:Simultaneous Quantification of Relevant Drugs and Metabolites in Blood Plasma
Source: Drug Test Anal. 2025 Jan 19;17(9):1502–15. doi: 10.1002/dta.3855 (PMC12401653; doi:10.1002/dta.3855)
Supplement: Supplementary file 1 — Table S1. Ionization effects using 1 mg/L solutions and internal standard diazepam‐d5. The table summarized the retention time (RT) of each analyte in minutes (min), the relative deviations of ionization in pooled solutions to single analyte solution, and the distribution of the analytes into three working solutions (1, 2, or 3). Acceptance criteria (AC) for ionization effects are ±25%. Values out of AC are marked in red. Table S2. Example table for the electronically stored five‐point calibration. Analytes are listed in alphabetical order. The table contains the name of the analytes according to the Toxtyper library, the internal standard (ISTD), the slope and intercept of the five‐point calibration curves, the limits of quantification (LLOQ and ULOQ), the concentration unit in ng/mL, the calibration concentration of the one‐point calibrator, the quantifier’s m/z value (Quan m/z), and the determined slope from an one‐point run (Cal_Slope_1). Table S3. Matrix effects (ME) with corresponding coefficient of variation (CV) for quality control sample (QC) Low and High. Acceptance criteria for ME were within ±30%. Table S4. Within‐run accuracy and precision data. Acceptance criteria (AC) for accuracy, ±30% of nominal value and AC precision, coefficient of variation (CV) < 30%. Values out of AC are marked in red. Table S5. Between‐run accuracy and precision data. Acceptance criteria (AC) for accuracy, ±30% of nominal value and AC precision, coefficient of variation (CV) < 30%. Values out of AC are marked in red. Table S6. Results of analyses of plasma samples analyzed by reference LC–MS/MS [1] or GC–MS [2] methods with interpretation of the values according to Schulz et al. [3]. Concentrations are given in ng/mL; not available, −. [file DTA-17-1502-s001.pdf]

Supplementary

Comprehensive Qualitative Drug Screening in Emergency Toxicology Using an Automated LC-MS^n System: Simultaneous Quantification of Relevant Drugs and Metabolites in Blood Plasma

Selina Hemmer, Maximilian Ninnig, Lea Wagmann, Sascha K. Manier, Markus R. Meyer *

Department of Experimental and Clinical Toxicology, Institute of Experimental and Clinical Pharmacology and Toxicology, Center for Molecular Signaling (PZMS), Saarland University, Homburg, Germany

*Corresponding author

Table S1. Ionization effects using 1 mg/L solutions and internal standard diazepam-d$_5$. The table summarized the retention time (RT) of each analyte in minutes (min), the relative deviations of ionization in pooled solutions to single analyte solution, and the distribution of the analytes into three working solutions (1, 2, or 3). Acceptance criteria (AC) for ionization effects are ±25 %. Values out of AC are marked in red.

Analyte	RT, min	Deviation, %	Working solution
Alprazolam	5.30	+5	1
Amisulpride	3.34	+11	3
Amitriptyline	4.84	+4	3
Aripiprazole	4.77	-40	1
Biperiden	4.65	+18	3
Bisoprolol	4.07	+16	1
Bromazepam	4.70	-36	2
Carbamazepine	4.97	-39	2
Chlorprothixene	5.04	+31	1
Citalopram	4.44	+14	1
Clobazam	5.67	+104	2
Clozapine	4.19	+95	3
Codeine	2.94	-11	3
Desipramine	4.70	+23	1
Diazepam	6.01	+7	3
Diazepam-M (nor-) / Nordiazepam	5.51	+54	3
Dihydrocodeine	2.90	+38	2
Diltiazem	4.51	+19	3
Diphenhydramine	4.40	+19	2
Doxepin	4.49	+11	2
Doxepin-M (nor-)	4.38	+10	1
Doxylamine	3.32	+29	2
Flupirtine	4.10	+27	2
Haloperidol	4.54	+14	1
Hydromorphone	2.64	+28	2
Imipramine	4.75	+28	3
Ketamine	3.44	+21	2
Levomepromazine	4.85	+20	1
Lorazepam	5.26	+23	3
Maprotiline	4.80	+75	2
Melperone	4.01	+15	2
Methadone	4.87	+126	3
Metoclopramide	3.50	+18	3
Metoprolol	3.73	-10	3
Mianserin	4.40	-29	3
Midazolam	4.37	+36	3
Mirtazapine	3.62	+28	2
Moclobemide	3.42	-9	1
Olanzapine	2.98	+24	1
Opipramol	4.34	+28	2
Oxazepam	5.16	+54	2
Oxcarbazepine	4.59	+14	3
Oxycodone	3.09	+46	2
Paracetamol	2.80	-3	3
Paroxetine	4.64	+10	2

Perazine	4.64	+51	1
Pethidine	3.87	+1	3
Pethidine-M (nor-)	3.84	+5	1
Pipamperone	3.32	+37	1
Promethazine	4.58	+37	2
Prothipendyl	4.40	+24	1
Quetiapine	4.33	+27	1
Ramipril	4.77	-26	2
Risperidone	3.97	+28	1
Risperidone-M (9-hydroxy-) / Paliperidone	3.88	-13	1
Sertraline	5.05	-24	2
Sulpiride	2.81	+59	1
Tapentadol	3.71	+56	3
Temazepam	5.58	+119	1
Tilidine	3.93	+8	3
Tilidine-M (nor-)	3.86	-9	2
Tramadol	3.66	+52	2
Tramadol-M (*O*-demethyl)	3.15	+23	3
Trimipramin-d3	4.97	+3	-
Venlafaxine	4.04	+7	3
Venlafaxine-M (*O*-demethyl)	3.66	+81	1
Verapamil	4.80	+29	1
Zolpidem	3.91	+33	2
Zopiclone	3.59	+2	1
Zuclopenthixol	5.02	+12	3

Table S2. Example table for the electronically stored five-point calibration. Analytes are listed in alphabetical order. The table contains the name of the analytes according to the Toxtyper library, the internal standard (ISTD), the slope and intercept of the five-point calibration curves, the limits of quantification (LLOQ and ULOQ), the concentration unit in ng/mL, the calibration concentration of the one-point calibrator, the quantifier's *m/z* value (Quan *m/z*), and the determined slope from an one-point run (Cal_Slope_1).

Analyte Name	ISTD	Slope	Intercept	LLOQ	ULOQ	Unit	Calibration Concentration	Quant *m/z*	Cal_Slope_1
9-Hydroxyrisperidone	D3-Trimipramine	2.00E-03	3.00E-03	100	2000	ng/ml	1000		2.00E-03
Alprazolam	D3-Trimipramine	2.58E-04	1.08E-02	50	500	ng/ml	250		2.58E-04
Amisulpride	D3-Trimipramine	1.64E-03	8.39E-03	100	2000	ng/ml	1000		1.64E-03
Amitriptyline	D3-Trimipramine	8.24E-04	2.60E-02	100	2000	ng/ml	1000		8.24E-04
Aripiprazole	D3-Trimipramine	4.04E-04	-2.77E-02	250	2000	ng/ml	1000		4.04E-04
Biperiden	D3-Trimipramine	1.68E-03	-3.40E-03	50	500	ng/ml	250		1.68E-03
Bisoprolol	D3-Trimipramine	1.11E-03	-1.09E-02	100	2000	ng/ml	1000		1.11E-03
Bromazepam	D3-Trimipramine	4.84E-05	-5.38E-02	1000	3000	ng/ml	2000	315.9	4.84E-05
Carbamazepine	D3-Trimipramine	1.08E-04	2.85E-01	2500	20000	ng/ml	10000		1.08E-04
Chlorprothixene	D3-Trimipramine	5.01E-04	-7.46E-02	250	2000	ng/ml	1000		5.01E-04
Citalopram	D3-Trimipramine	1.46E-03	-4.03E-02	100	2000	ng/ml	1000		1.46E-03
Clobazam	D3-Trimipramine	1.61E-04	1.67E-03	100	2000	ng/ml	1000	301.1	1.61E-04
Clozapine	D3-Trimipramine	1.08E-03	-9.52E-03	100	2000	ng/ml	1000		1.08E-03
Codeine	D3-Trimipramine	4.37E-04	-3.37E-03	250	2000	ng/ml	1000		4.37E-04
Desipramine	D3-Trimipramine	8.02E-04	-1.30E-01	250	2000	ng/ml	1000		8.02E-04
Desmethylvenlafaxine	D3-Trimipramine	7.74E-04	-4.20E-02	100	2000	ng/ml	1000		7.74E-04
Diazepam	D3-Trimipramine	3.95E-04	-2.68E-02	500	3000	ng/ml	1500		3.95E-04
Dihydrocodeine	D3-Trimipramine	6.83E-04	-3.85E-02	250	2000	ng/ml	1000		6.83E-04
Diltiazem	D3-Trimipramine	1.73E-03	-5.17E-02	100	2000	ng/ml	1000		1.73E-03
Diphenhydramine	D3-Trimipramine	8.76E-04	-8.83E-03	500	2500	ng/ml	1500		8.76E-04
Doxepin	D3-Trimipramine	9.17E-04	-3.01E-03	100	2000	ng/ml	1000		9.17E-04
Doxylamine	D3-Trimipramine	5.58E-04	2.23E-02	200	2000	ng/ml	1000		5.58E-04
Flupirtine	D3-Trimipramine	4.75E-04	-5.46E-02	1000	3000	ng/ml	2000	305.14	4.75E-04
Haloperidol	D3-Trimipramine	1.32E-03	-1.32E-02	50	500	ng/ml	250		1.32E-03
Hydromorphone	D3-Trimipramine	2.05E-04	-8.86E-04	50	500	ng/ml	250	286.1	2.05E-04
Imipramine	D3-Trimipramine	9.92E-04	-3.16E-02	250	2000	ng/ml	1000		9.92E-04
Ketamine	D3-Trimipramine	2.53E-04	-1.78E-02	1000	10000	ng/ml	5000		2.53E-04
Levomepromazine	D3-Trimipramine	9.97E-04	-2.87E-02	200	2000	ng/ml	1000		9.97E-04

Lorazepam	D3-Trimipramine	5.26E-05	-3.94E-03	500	2500	ng/ml	1500		320.94	5.26E-05
Maprotiline	D3-Trimipramine	4.55E-04	1.31E-02	100	2000	ng/ml	1000			4.55E-04
Melperone	D3-Trimipramine	6.03E-04	-3.80E-02	1000	2000	ng/ml	1000			6.03E-04
Methadone	D3-Trimipramine	8.25E-04	-8.78E-03	100	2000	ng/ml	1000			8.25E-04
Metoclopramide	D3-Trimipramine	8.82E-04	-8.36E-03	100	2000	ng/ml	1000			8.82E-04
Metoprolol	D3-Trimipramine	6.54E-04	2.02E-02	100	2000	ng/ml	1000			6.54E-04
Mianserin	D3-Trimipramine	5.62E-04	4.97E-02	50	500	ng/ml	250			5.62E-04
Midazolam	D3-Trimipramine	1.04E-03	-3.05E-03	100	2000	ng/ml	1000			1.04E-03
Mirtazapine	D3-Trimipramine	6.41E-04	-6.12E-03	150	2000	ng/ml	1000			6.41E-04
Moclobemide	D3-Trimipramine	3.51E-04	-6.30E-03	500	5000	ng/ml	2500			3.51E-04
Nordiazepam	D3-Trimipramine	1.06E-04	3.85E-02	750	2500	ng/ml	1500		271.1	1.06E-04
Nordoxepin	D3-Trimipramine	6.19E-04	-1.21E-03	50	500	ng/ml	250			6.19E-04
Norpethidine	D3-Trimipramine	4.75E-04	1.43E-03	50	500	ng/ml	250			4.75E-04
Nortilidine	D3-Trimipramine	5.16E-04	3.36E-02	100	2000	ng/ml	1000			5.16E-04
O-Desmethyltramadol	D3-Trimipramine	4.72E-04	8.42E-03	100	2000	ng/ml	1000			4.72E-04
Olanzapine	D3-Trimipramine	8.08E-04	-4.20E-03	50	500	ng/ml	250			8.08E-04
Opipramol	D3-Trimipramine	1.62E-03	-5.83E-02	100	2000	ng/ml	1000			1.62E-03
Oxazepam	D3-Trimipramine	7.66E-05	-4.46E-02	1000	3000	ng/ml	2000		287.06	7.66E-05
Oxcarbazepine	D3-Trimipramine	5.45E-05	8.24E-02	2500	65000	ng/ml	20000		253.1	5.45E-05
Oxycodone	D3-Trimipramine	3.51E-04	4.31E-02	250	2000	ng/ml	1000			3.51E-04
Paracetamol	D3-Trimipramine	4.46E-06	3.09E-02	2500	120000	ng/ml	60000		152.07	4.46E-06
Paroxetine	D3-Trimipramine	4.41E-04	2.28E-03	50	500	ng/ml	250			4.41E-04
Perazine	D3-Trimipramine	6.40E-04	-4.62E-02	100	2000	ng/ml	1000			6.40E-04
Pethidine	D3-Trimipramine	5.32E-04	4.66E-02	500	2500	ng/ml	1500			5.32E-04
Pipamperone	D3-Trimipramine	9.81E-04	-1.76E-02	250	2000	ng/ml	1000			9.81E-04
Promethazine	D3-Trimipramine	4.91E-04	2.99E-02	100	2000	ng/ml	1000			4.91E-04
Prothipendyl	D3-Trimipramine	7.28E-04	-4.29E-03	50	500	ng/ml	250			7.28E-04
Quetiapine	D3-Trimipramine	1.65E-03	-2.32E-02	100	2000	ng/ml	1000			1.65E-03
Ramipril	D3-Trimipramine	1.71E-04	1.79E-03	100	500	ng/ml	250			1.71E-04
Risperidone	D3-Trimipramine	2.39E-03	-1.35E-02	50	500	ng/ml	250			2.39E-03
Sertraline	D3-Trimipramine	5.00E-05	3.48E-02	250	2000	ng/ml	1000			5.00E-05
Sulpiride	D3-Trimipramine	3.82E-04	-3.29E-02	500	2500	ng/ml	1500		342.15	3.82E-04
Tapentadol	D3-Trimipramine	5.50E-04	7.02E-03	100	2000	ng/ml	1000			5.50E-04
Temazepam	D3-Trimipramine	1.31E-04	-7.97E-04	500	2000	ng/ml	1500			1.31E-04

Tilidine	D3-Trimipramine	7.95E-04	1.63E-02	100	2000	ng/ml	1000		7.95E-04
Tramadol	D3-Trimipramine	6.64E-04	8.79E-02	250	2000	ng/ml	1000	264.2	6.64E-04
Venlafaxine	D3-Trimipramine	1.18E-03	9.11E-03	100	2000	ng/ml	1000		1.18E-03
Verapamil	D3-Trimipramine	2.29E-03	-7.27E-02	100	2000	ng/ml	1000		2.29E-03
Zolpidem	D3-Trimipramine	1.65E-03	-3.49E-02	100	2000	ng/ml	1000		1.65E-03
Zopiclone	D3-Trimipramine	3.27E-04	-1.19E-04	100	2000	ng/ml	1000	389.1	3.27E-04
Zuclopenthixol	D3-Trimipramine	4.94E-04	-1.12E-02	50	500	ng/ml	250		4.94E-04

Table S3. Matrix effects (ME) with corresponding coefficient of variation (CV) for quality control sample (QC) Low and High. Acceptance criteria for ME were within ±30 %.

**Analyte**	**ME (CV), %**	
	**QC Low**	**QC High**
Alprazolam	123 (13)	125 (16)
Amisulpride	105 (12)	111 (10)
Amitriptyline	100 (5)	105 (7)
Aripiprazole	108 (16)	119 (29)
Biperiden	114 (13)	106 (11)
Bisoprolol	119 (11)	124 (17)
Bromazepam	111 (10)	122 (9)
Carbamazepine	116 (9)	108 (8)
Chlorprothixene	101 (10)	105 (11)
Citalopram	110 (10)	120 (17)
Clobazam	100 (10)	116 (10)
Clozapine	106 (12)	115 (25)
Codeine	102 (4)	115 (14)
Desipramine	111 (15)	109 (15)
Diazepam	112 (3)	110 (11)
Diazepam-M (nor-) / Nordiazepam	112 (8)	117 (17)
Dihydrocodeine	112 (13)	116 (8)
Diltiazem	118 (8)	121 (15)
Diphenhydramine	114 (7)	116 (5)
Doxepin	110 (6)	113 (6)
Doxepin-M (nor-)	114 (18)	110 (9)
Doxylamine	114 (8)	116 (9)
Flupirtine	110 (4)	115 (7)
Haloperidol	118 (11)	116 (18)
Hydromorphone	104 (13)	111 (10)
Imipramine	110 (5)	109 (10)
Ketamine	109 (9)	113 (6)
Levomepromazine	104 (11)	112 (11)
Lorazepam	128 (6)	115 (11)
Maprotiline	98 (3)	104 (6)
Melperone	113 (9)	112 (7)
Methadone	117 (10)	116 (13)
Metoclopramide	108 (7)	108 (6)
Metoprolol	117 (7)	116 (8)
Mianserin	108 (10)	116 (9)
Midazolam	111 (9)	112 (9)
Mirtazapine	108 (15)	117 (12)
Moclobemide	121 (8)	116 (6)
Olanzapine	122 (19)	126 (8)
Opipramol	89 (7)	107 (17)
Oxazepam	129 (6)	126 (11)
Oxcarbazepine	117 (5)	112 (12)
Oxycodone	110 (14)	111 (9)
Paracetamol	116 (4)	106 (15)
Paroxetine	95 (10)	105 (7)
Perazine	82 (15)	84 (15)
Pethidine	110 (7)	115 (10)
Pethidine-M (nor-)	107 (9)	116 (16)

Pipamperone	110 (11)	124 (20)
Promethazine	112 (11)	109 (8)
Prothipendyl	105 (9)	119 (14)
Quetiapine	113 (16)	125 (11)
Ramipril	117 (12)	129 (6)
Risperidone	119 (11)	125 (13)
Risperidone-M (9-hydroxy-) / Paliperidone	122 (9)	126 (13)
Sertraline	92 (17)	93 (12)
Sulpiride	118 (12)	113 (13)
Tapentadol	120 (5)	115 (12)
Temazepam	120 (15)	129 (13)
Tilidine	112 (13)	113 (9)
Tilidine-M (nor-)	107 (11)	111 (12)
Tramadol	114 (12)	115 (6)
Tramadol-M (*O*-demethyl-)	122 (7)	110 (13)
Venlafaxine	117 (7)	112 (5)
Venlafaxine-M (*O*-demethyl-)	112 (13)	117 (7)
Verapamil	118 (8)	112 (7)
Zolpidem	117 (8)	110 (8)
Zopiclone	120 (17)	119 (12)
Zuclopenthixol	91 (33)	71 (25)

Table S4. Within-run accuracy and precision data. Acceptance criteria (AC) for accuracy, ±30 % of nominal value and AC precision, coefficient of variation (CV) < 30 %. Values out of AC are marked in red.

**Analyte**	**LLOQ**		**QC LOW**		**QC High**	
	**Accuracy, %**	**Precision, %**	**Accuracy, %**	**Precision, %**	**Accuracy, %**	**Precision, %**
Alprazolam	108	16	127	12	130	13
Amisulpride	83	13	85	13	83	3
Amitriptyline	110	11	111	14	87	8
Aripiprazole	208	24	253	28	170	37
Biperiden	94	15	114	15	71	5
Bisoprolol	86	20	101	26	99	10
Bromazepam	83	17	91	22	112	12
Carbamazepine	90	18	97	22	87	8
Chlorprothixene	101	15	105	11	94	11
Citalopram	84	23	115	20	124	4
Clobazam	116	7	111	17	99	8
Clozapine	91	10	107	5	80	12
Codeine	127	11	121	13	98	8
Desipramine	93	26	90	19	98	8
Diazepam	118	10	109	9	102	6
Diazepam-M (nor-) / Nordiazepam	70	1	125	13	129	4
Dihydrocodeine	114	6	122	19	96	10
Diltiazem	122	12	129	7	104	7
Diphenhydramine	105	39	92	32	95	39
Doxepin	71	8	74	21	83	6
Doxepin-M (nor-)	81	30	124	19	83	5
Doxylamine	-	-	105	23	77	26
Flupirtine	81	10	83	19	85	10
Haloperidol	98	15	111	16	120	6
Hydromorphone	70	11	87	18	76	10
Imipramine	118	8	108	11	98	13

Ketamine	68	46	74	61	87	26
Levomepromazine	76	20	92	14	97	5
Lorazepam	111	7	102	13	129	7
Maprotiline	81	27	101	16	98	10
Melperone	55	50	72	57	85	38
Methadone	89	11	96	13	85	20
Metoclopramide	102	10	121	13	98	5
Metoprolol	97	11	114	18	83	9
Mianserin	98	16	115	10	88	12
Midazolam	109	5	128	11	96	10
Mirtazapine	98	7	90	19	79	5
Moclobemide	83	28	87	29	101	10
Olanzapine	95	13	108	17	114	13
Opipramol	102	20	100	21	81	8
Oxazepam	73	13	80	15	109	6
Oxcarbazepine	75	18	90	25	70	6
Oxycodone	95	9	93	19	82	5
Paracetamol	81	8	110	13	85	5
Paroxetine	92	30	101	28	109	7
Perazine	78	25	93	21	115	10
Pethidine	83	40	82	47	74	46
Pethidine-M (nor-)	72	20	78	22	91	13
Pipamperone	83	24	89	18	115	9
Promethazine	99	17	101	8	100	8
Prothipendyl	70	17	79	20	114	9
Quetiapine	79	27	87	22	115	8
Ramipril	129	8	126	11	86	13
Risperidone	76	15	82	14	98	6
Risperidone-M (9-hydroxy-) / Paliperidone	101	9	113	15	112	11
Sertraline	159	71	141	50	102	23
Sulpiride	91	18	76	12	108	8
Tapentadol	113	12	124	19	78	19

Temazepam	116	12	125	19	121	7
Tilidine	92	30	95	35	66	44
Tilidine-M (nor-)	61	130	64	121	110	59
Tramadol	84	25	77	26	73	27
Tramadol-M (*O*-demethyl-)	108	10	127	19	87	5
Venlafaxine	102	9	107	15	82	12
Venlafaxine-M (*O*-demethyl-)	70	26	81	20	97	8
Verapamil	75	14	90	22	101	9
Zolpidem	125	15	134	19	98	10
Zopiclone	70	19	71	15	71	9
Zuclopenthixol	102	20	97	17	116	8

Table S5. Between-run accuracy and precision data. Acceptance criteria (AC) for accuracy, ±30 % of nominal value and AC precision, coefficient of variation (CV) < 30 %. Values out of AC are marked in red.

**Analyte**	**LLOQ**		**QC LOW**		**QC High**	
	**Accuracy, %**	**Precision, %**	**Accuracy, %**	**Precision, %**	**Accuracy, %**	**Precision, %**
Alprazolam	100	28	99	21	117	13
Amisulpride	93	15	93	15	84	12
Amitriptyline	102	15	121	11	93	10
Aripiprazole	200	38	199	38	143	36
Biperiden	98	16	124	14	87	15
Bisoprolol	86	23	88	25	90	12
Bromazepam	104	21	109	18	110	12
Carbamazepine	98	28	92	28	87	14
Chlorprothixene	100	20	100	24	92	8
Citalopram	110	30	118	20	116	9
Clobazam	114	18	117	13	102	10
Clozapine	107	16	119	15	80	10
Codeine	122	15	122	15	98	8
Desipramine	99	24	90	22	97	8
Diazepam	120	8	115	12	99	7
Diazepam-M (nor-) / Nordiazepam	115	29	129	9	115	12
Dihydrocodeine	116	17	118	19	113	21
Diltiazem	117	12	130	7	102	9
Diphenhydramine	38	368	65	84	112	35
Doxepin	70	18	72	23	81	7
Doxepin-M (nor-)	101	30	118	19	97	21
Doxylamine	-	-	98	23	81	28
Flupirtine	107	21	104	19	93	9
Haloperidol	94	22	102	18	110	11
Hydromorphone	73	15	77	18	82	13
Imipramine	110	11	111	10	97	10

Ketamine	40	319	59	131	88	30
Levomepromazine	87	16	88	13	98	10
Lorazepam	107	7	97	9	114	12
Maprotiline	109	26	129	24	95	13
Melperone	72	67	80	49	95	30
Methadone	74	23	93	15	84	12
Metoclopramide	98	11	109	14	90	9
Metoprolol	88	18	98	21	86	11
Mianserin	80	23	107	11	83	14
Midazolam	116	12	127	12	98	8
Mirtazapine	92	14	87	17	83	11
Moclobemide	82	22	79	27	92	14
Olanzapine	51	139	94	85	135	42
Opipramol	98	22	105	18	87	15
Oxazepam	99	22	98	16	101	10
Oxcarbazepine	70	22	93	25	70	8
Oxycodone	103	16	98	15	86	12
Paracetamol	104	30	101	23	85	16
Paroxetine	113	25	122	21	97	15
Perzine	77	25	85	26	97	23
Pethidine	-654	-114	110	785	130	255
Pethidine-M (nor-)	71	45	79	46	92	22
Pipamperone	84	30	88	28	102	15
Promethazine	104	14	117	18	103	8
Prothipendyl	83	29	85	26	99	14
Quetiapine	87	28	96	28	96	17
Ramipril	111	28	117	20	100	27
Risperidone	79	22	80	18	81	29
Risperidone-M (9-hydroxy-) / Paliperidone	104	26	110	17	98	18
Sertraline	113	66	127	62	70	55
Sulpiride	97	29	86	25	97	14
Tapentadol	93	27	127	19	92	19

Temazepam	113	14	105	12	115	8
Tilidine	36	611	71	181	70	89
Tilidine-M (nor-)	-9	-2266	28	426	111	49
Tramadol	91	30	75	26	77	21
Tramadol-M (*O*-demethyl)	117	14	128	15	89	9
Venlafaxine	85	25	114	20	89	11
Venlafaxine-M (*O*-demethyl)	78	30	81	25	88	12
Verapamil	83	27	89	25	95	10
Zolpidem	121	21	125	18	98	10
Zopiclone	76	20	74	17	83	17
Zuclopenthixol	104	19	106	16	101	15

Table S6. Results of analyses of plasma samples analyzed by reference LC-MS/MS [1] or GC-MS [2] methods with interpretation of the values according to Schulz et al. [3]. Concentrations are given in ng/mL; not available, -.

Case	Analyte	LLOQ	Calculated Conc.	Target Value		Interpretation
				GC-MS	LC-MS	
1	Doxepin	100	173	<300	-	therapeutic
	Levomepromazine	200	927	-	-	toxic
	Quetiapine	100	1,826	-	2,170	toxic
2	Diazepam	500	2,900	2,100	-	overdosed
	Diazepam-M (nor-) / Nordiazepam	750	1,200	700	-	
3	Opipramol	100	1,968	-	2,500	toxic
4	Bisoprolol	100	<100	-	10	therapeutic
	Doxepin	100	<100	<300	-	therapeutic
	Methadone	100	548	500	-	therapeutic
5	Lorazepam	500	618	-	930	toxic
6	Amitriptyline	100	122	<200	160	therapeutic
	Bisoprolol	100	<100	-	<10	therapeutic
	Oxycodone	250	<250	-	70	therapeutic
	Promethazine	100	<100	-	90	therapeutic
7	Promethazine	100	<100	-	20	therapeutic
	Risperidone	50	<50	-	6	therapeutic
	Risperidone-M (9-hydroxy-) / Paliperidone	100	<100	-	10	therapeutic
	Venlafaxine	100	422	-	300	overdosed
	Venlafaxine-M (*O*-demethyl)	100	176	-	200	
8	Amitriptyline	100	<100	<200	-	therapeutic
	Tilidine	100	<100	-	<50	therapeutic
	Tilidine-M (nor-)	100	278	-	200	-
9	Diazepam	500	<500	<1,000	-	therapeutic
	Diazepam-M (nor-) / Nordiazepam	750	<750	<1,000	-	

	Olanzapine	50	80	-	40	therapeutic
	Paracetamol	2,500	<2,500	-	<2,500	(sub-)therapeutic
	Quetiapine	100	<100	-	<100	(sub-)therapeutic
	Risperidone-M (9-hydroxy-) / Paliperidone	100	175	-	150	overdosed
10	Zopiclone	100	1,134	-	900	toxic
11	Prothipendyl	50	440	-	600	toxic
12	Amitriptyline	100	100	-	100	therapeutic
13	Tramadol	250	415	-	500	therapeutic
	Tramadol-M (*O*-demethyl-)	100	<100	-	40	-
	Paracetamol	2,500	<2,500	-	-	(sub-)therapeutic
14	Mirtazapine	150	591	500	-	overdosed
	Quetiapine	100	926	-	790	overdosed
15	Bisoprolol	100	<100	-	20	therapeutic
	Venlafaxine	100	<100	-	100	therapeutic
	Venlafaxine-M (*O*-demethyl-)	100	227	-	250	
16	Amisulpride	100	293	-	320	therapeutic
	Diazepam	500	1,003	<1,000	-	therapeutic
	Diazepam-M (nor-) / Nordiazepam	750	1,333	1,400	-	
17	Alprazolam	77	77	-	60	overdosed
	Citalopram	100	<100	<200	-	therapeutic
	Mirtazapine	150	<150	100	-	therapeutic
18	Doxepin	100	1,062	1,500	-	toxic
	Doxepin-M (nor-)	50	240	-	-	-
	Midazolam	100	<100	<100	-	therapeutic
	Paracetamol	2,500	3,5780	-	-	overdosed
19	Opipramol	100	1,909	-	2,300	toxic
	Oxazepam	1,000	>3,000	-	2,800	toxic
20	Olanzapine	50	59	-	50	therapeutic
21	Bisoprolol	100	<100	-	-	therapeutic
	Zolpidem	100	708	700	-	toxic
22	Bromazepam	1,000	1,184	-	970	toxic

23	Zolpidem	100	554	-	500	toxic
24	Opipramol	100	1,968	-	2,500	toxic
25	Midazolam	100	1,726	1,200	-	toxic
26	Carbamazepine	2,500	7,300	-	9,500	therapeutic
	Quetiapine	100	<100	-	<100	(sub-)therapeutic
	Risperidone-M (9-hydroxy-) / Paliperidone	100	<100	-	6	(sub-)therapeutic
	Zuclopenthixol	50	<50	-	20	therapeutic
27	Diazepam	500	<500	-	-	therapeutic
	Diazepam-M (nor-) / Nordiazepam	750	<750	-	-	
	Olanzapine	50	80	-	40	therapeutic
	Paracetamol	2,500	<2,500	-	-	(sub-)therapeutic
	Quetiapine	100	<100	-	<100	(sub-)therapeutic
	Risperidone-M (9-hydroxy-) / Paliperidone	100	175	-	150	overdosed
28	Zopiclone	100	1134	-	900	toxic
29	Biperiden	50	<50	-	-	toxic
	Diazepam	1,370	1,370	1,000	-	overdosed
	Diazepam-M (nor-) / Nordiazepam	>2,500	>2500	1,800	-	
	Methadone	1,248	1,248	<1,000	-	toxic
	Zuclopenthixol	50	50	-	-	therapeutic

References

1. Michely, J.A. and H.H. Maurer, A multi-analyte approach to help in assessing the severity of acute poisonings - Development and validation of a fast LC-MS/MS quantification approach for 45 drugs and their relevant metabolites with one-point calibration, *Drug Test Anal* 10(1) (2018) 164-176. https://doi.org/10.1002/dta.2257.
2. Meyer, G.M., A.A. Weber, and H.H. Maurer, Development and validation of a fast and simple multi-analyte procedure for quantification of 40 drugs relevant to emergency toxicology using GC-MS and one-point calibration, *Drug Test Anal* 6(5) (2014) 472-81. https://doi.org/10.1002/dta.1555.
3. Schulz, M., et al., Revisited: Therapeutic and toxic blood concentrations of more than 1100 drugs and other xenobiotics, *Crit Care* 24(1) (2020) 195. https://doi.org/10.1186/s13054-020-02915-5.
